# Lung Ultrasound in Hemodialysis Patients—Which Protocol Is More Accurate and Informative in Daily Clinical Practice: A Systematic Review

**DOI:** 10.3390/life15020272

**Published:** 2025-02-11

**Authors:** Christodoulos Keskinis, Konstantina Bacharidou, Stylianos Panagoutsos, Efstathios Mitsopoulos

**Affiliations:** 1 Department of Nephrology, Papageorgiou Hospital, 56429 Thessaloniki, Greece; mitsopouloss@yahoo.com; 2 1st Department of Internal Medicine, Papageorgiou Hospital, 56429 Thessaloniki, Greece; kwnstantina-mp@hotmail.com; 3 Department of Nephrology, Medical School, Democritus University of Thrace, 68100 Alexandroupolis, Greece; spanagou@med.duth.gr

**Keywords:** hemodialysis, lung ultrasound, protocols, scanning zones, hypervolemia, lung congestion, 28 zone, 16 zone, 12 zone, 8 zone, 6 zone, 4 zone

## Abstract

Lung ultrasound can detect hidden lung congestion in hemodialysis (HD) patients, even though they present no symptoms of hypervolemia. The 28-zone protocol is the one mainly assessed in the majority of studies. However, it is quite time consuming, making its integration into daily clinical practice difficult. Alternative approaches have been proposed that require fewer scanning zones. This systematic review used various combinations of the following keywords: “Lung ultrasound”, “Hemodialysis”, “Scanning protocols”, “Scanning zones”, “28-points”, and “28-zones” via PubMed’s and Google Scholar’s search engines. Six relevant studies were obtained, five of which refer to the adult population and one to children. Initially, the first published study compared the 28-zone protocol to the 8-zone protocol, while three studies compared the 28-zone protocol to the 8-zone, 6-zone, and 4-zone protocols. Another study compared the 16-zone protocol to the 12-zone and 8-zone protocols. Finally, one study compared the 28-zone protocol to the 8-zone protocol in children. There are several alternatives in the literature for applying abbreviated versions of the 28-zone protocol to save time in daily clinical practice. However, none of these protocols provide the same accuracy as the 28-zone protocol. Therefore, it should be preferred when the clinical question regarding pulmonary congestion remains. Further multicenter studies are required to determine whether any shorter version of the 28-zone protocol can sufficiently replace it in daily clinical practice.

## 1. Introduction

Hypervolemia is a frequent issue in end-stage kidney disease (ESKD) patients requiring HD and it is associated with hypertension, cardiovascular (CV) events, and mortality [1,2,3,4,5,6,7]. Ensuring proper water and sodium balance is both the most important and the most challenging aspect of management [3,8]. Significant improvement in hypertension and echocardiographic parameters following the correction of imbalance between fluid intake and removal has been well established [9]. To date, there is no exact method to determine the amount of ultrafiltration required in each dialysis session [2,3,10]. As a result, nephrologists use the terms “dry weight” (DW) or “ideal weight” (ID) to estimate a patient’s optimal body weight [10,11]. Ultrafiltration is then applied based on this weight. However, this approach has several limitations, as many physicians or nurses typically consider the difference between the admission weight and the dry weight to represent the target ultrafiltration, often relying on arbitrary judgments.

The term DW has been modified multiple times since its initial introduction in 1967 by Thomson et al. [12]. Firstly, it was defined as the reduction in blood pressure to adequately low levels due to hyperfiltration once other causes of hypotension had been excluded [12]. Later, Henderson clarified that DW is determined by the loss of body weight that is regained between dialysis sessions, with its loss often leading to hypotensive episodes [13]. In 1996, Charra et al. proposed another definition, stating that DW is the body weight at the end of a hemodialysis session that allows the patient to maintain normal blood pressure until the next session, regardless of salt intake and without the need for anti-hypertensive medication [14]. Today, the most widely accepted definition, proposed in 2009 by Sinha and Agarwal, is that of dry weight as the minimum possible body weight at the end of a hemodialysis session, with which the patient experiences the least symptoms of hypo- and hypervolemia during the next session [15].

Clinical examination, which involves checking for lung crackles, assessing pedal edema, and observing jugular vein distension, combined with blood pressure measurements, is commonly used to evaluate DW [2]. However, plenty of nephrologists are quite skeptical about detecting hypervolemia through physical examination since clinical evaluation has consistently shown limited diagnostic accuracy in detecting fluid accumulation in HD patients [16,17]. To enhance the accuracy of DW assessment, various diagnostic techniques have been developed, such as bioimpedance methods, chest X-rays, using biochemical markers (e.g., BNP/NT-proBNP), performing echocardiography (e.g., left ventricular [LV] filling pressures and the E/e’ ratio), and measuring the diameter of the inferior vena cava [2]. These methods are widely utilized and provide reliable evaluations of DW.

**Figure 1:**
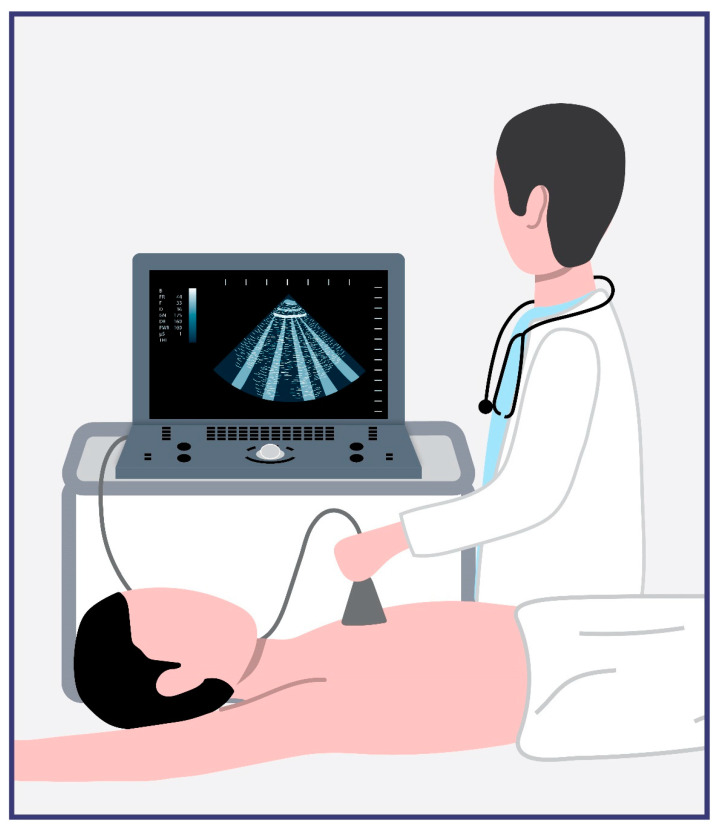
Most examiners begin the LUS assessment with the patient in a supine position.

In the past decade, a new technique has emerged to assess the DW of HD patients: lung ultrasound (LUS) [18]. LUS cannot provide important information on healthy individuals, although it can detect fluid in the extracellular interstitial lung tissue. Zoccali and Mallamaci first identified the interstitial lung tissue as a compartment that may reflect the total extracellular fluid volume, allowing for potential interventions [19]. A potential hidden pulmonary congestion can be identified and treated with this technique. LUS is an easy-to-perform, non-invasive, and radiation-free method that is both quick and simple to execute [11]. Although it was previously overlooked due to the lung being an air-filled organ, this initial limitation can be turned into an advantage in pathological conditions where air content in the lung is reduced and fluid materials with higher density, such as extravascular water, are present [2,20]. In such cases, the ultrasound beam partially reflects in the deeper regions of the lung, creating vertical lines known as B-lines, otherwise called comets due to their shape [20]. All common probe types (array, convex, and microconvex) can be used, while the frequencies may range from 2.5 MHz to 12.5 MHz [2,21,22]. The exam initiates when the probe is placed between the ribs, avoiding direct contact with the ribs themselves. Lung congestion is initially assessed by placing the patient in a supine position to examine the anterior chest [2]. Then, the patient is repositioned in a semi-supine position. The right axillary lines are scanned first, followed by the left. An example of the patient’s position is presented in Figure 1.

Different scanning protocols have been applied to detect pulmonary congestion as an immediate marker of hypervolemia, with all protocols focusing on scanning the anterior and lateral chest regions. An increased B-line score is correlated with hypervolemia and lung congestion, while a low pre-dialytic B-line score has been conversely associated with intradialytic hypotensive episodes [23]. The original 28-zone scanning protocol, introduced by Jambrik, is the most established and widely used protocol in the literature [24].

Given the time-consuming nature of this examination, there is a growing need to identify an abbreviated version that maintains accuracy [25,26]. The most common alternatives in the literature involve the scanning of eight, six, and four sites, corresponding to the 8-zone, 6-zone, and 4-zone, respectively. These protocols evaluate the same zones on each hemithorax.

**Figure 2:**
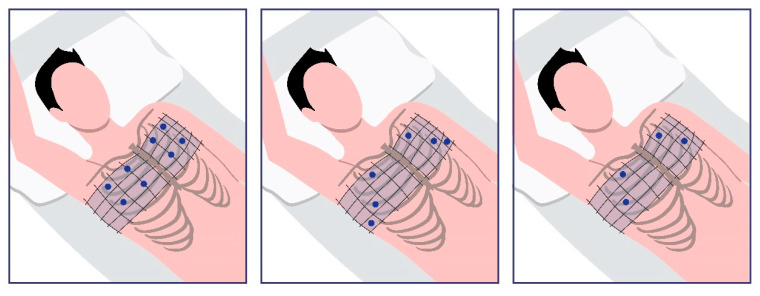
The most commonly reported alternatives to the 28-zone protocol are the 8-zone, 6-zone, and 4-zone scanning protocols. All of them involve scanning the same specific sites in each hemithorax.

The 8-zone protocol, proposed by Volpicelli, involves scanning two anterior sites (located between the sternum and the anterior axillary line) and two lateral sites (located between the anterior and posterior axillary lines) on each side of the chest [1,2,27]. The 6-zone protocol includes specific intercostal spaces, such as the second at the midclavicular line, the fourth at the anterior axillary line, and the fifth at the midaxillary line, which are scanned on both sides. The cut-off for hypervolemia using the 6-zone protocol varies in the literature; it may be defined as the detection of three B-lines at one site in each hemithorax or the detection of three B-lines at two sites in each hemithorax [1,2]. The 4-zone protocol includes scanning the second intercostal space at the midclavicular line and the fourth intercostal space at the anterior axillary line on both sides [2,26]. The 8-zone, 6-zone and 4-zone protocols are depicted in the following Figure 2.

**Figure 3:**
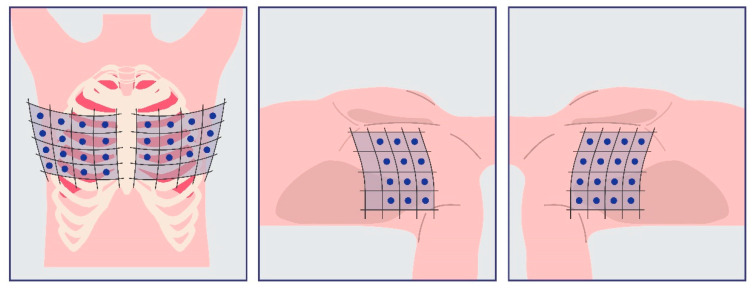
The 28-zone protocol is the most commonly evaluated in the literature. It can also classify the degree of hypervolemia and is the only protocol reported that requires a different number of zones to be scanned in each hemithorax.

On the other hand, the 28-zone protocol, which is most frequently used in hemodialysis HD patients, scans a total of 28 anatomical sites across both lung fields. This includes the second–fifth intercostal spaces on the right side, covering the parasternal line, interclavicular line, anterior axillary line, and mid-axillary line (16 points in total), and the second–fourth intercostal spaces on the left side, covering the parasternal line, interclavicular line, anterior axillary line, and mid-axillary line (12 points in total) [1,2]. In this protocol, up to 10 B-lines may be detected at each anatomical location, with a total possible maximum of 280 B-lines [11]. This is the only protocol that does not require scanning the exact same sites on both hemithoraxes as depicted in the following Figure 3.

There is ongoing debate about which protocol is most suitable for assessing DW in HD patients [25,26]. The 28-zone protocol is clearly time consuming, which makes it challenging to apply regularly. Therefore, it would be interesting to know whether protocols with fewer points offer the same level of accuracy or if they could miss signs of concealed lung congestion. If they do offer the same level of accuracy, then LUS could be integrated into the daily clinical practice of dialysis units, providing crucial information regarding the hydration status of HD patients. In this review, we aim to examine the available literature comparing the 28-point protocol with those that involve fewer points.

## 2. Materials and Methods

**Figure 4:**
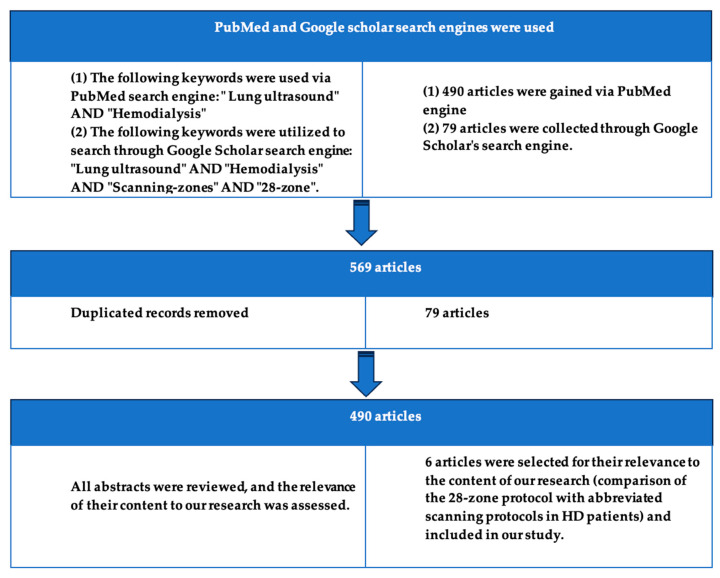
Our research flow diagram is illustrated below. Six articles were ultimately included in our study due to their relevance to the comparison between the 28-zone protocol and abbreviated scanning protocols.

Our research was carried out by utilizing the PubMed and Google Scholar search engines. We performed multiple searches using various combinations of the following keywords: “Lung ultrasound”, “Hemodialysis”, “Scanning protocols”, “Scanning zones”, “28-points”, and “28-zones”. In total, we reviewed the abstracts of over 400 articles related to these topics. After carefully examining the available literature, we selected only those studies that specifically compared the 28-zone protocol with other protocols discussed in the literature. These other protocols require fewer anatomical regions of the chest wall to be scanned. After narrowing down the list based on these criteria, 6 articles were included in our review. Two reviewers independently assessed the Cochrane Risk of Bias (RoB) for each included study [28]. Our study was conducted according to the PRISMA guidelines, and the research methodology is depicted in the following flow diagram, Figure 4 [29].

## 3. Results

We subdivided the articles we found according to their content. The first article published includes the comparison of the 28-zone protocol with the 8-zone protocol [25]. The following three articles compare the 28-zone protocol with the most commonly recorded protocols in the literature [1,11,30,31]. The fifth article included chronic kidney disease patients (CKD), mainly HD patients, who underwent 16-zone, 12-zone, and 8-zone LUS scanning protocols [32]. Finally, one study referring to the pediatric population has been included [33].

Only six studies have compared the 28-zone protocol with other existing scanning protocols trying to answer the following question: “Do the protocols requiring fewer scanning zones provide similar clinical data to the nephrologists?” All these articles have been published since 2020, indicating the existing interest among nephrologists in this topic.

### 3.1. Articles Including the Comparison of the 28-Zone Protocol with the Corresponding 8-Zone Protocol in Adult HD Patients

**Figure 5:**
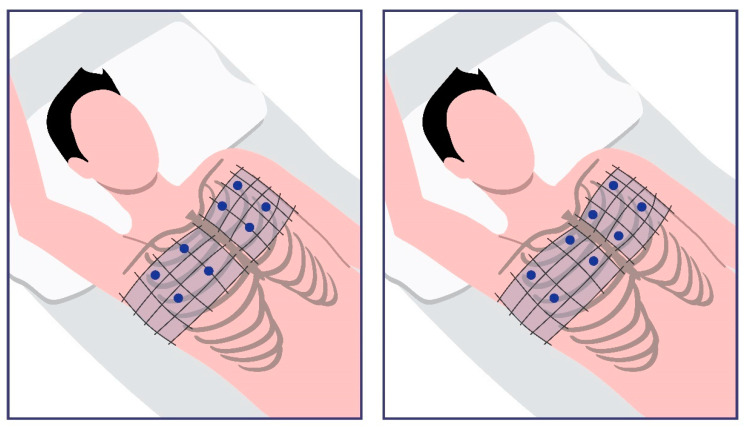
Volpicelli’s protocol is presented on the left. It is the main 8-zone scanning protocol reported in the literature. Torino et al. [25] presented a different 8-zone scanning protocol, which was partially derived from the full 28-zone scanning protocol.

Torino et al. published the first study that compared the 28-zone protocol to the 8-zone protocol [25]. This study included the most HD patients (303) evaluated with LUS so far. All participants were scanned with both protocols before their scheduled session during the mid-week HD of their tri-weekly program. All the measurements were conducted by five different examiners with various experience. In fact, only two of them were familiar with the technique. Over an average follow-up period of 3.0 years (IQR 1.9–4.5 years), both protocols were highly correlated, with a Spearman correlation coefficient of 0.93, and their predictive value was assessed for overall mortality and cardiovascular events. A univariate analysis was performed and exhibited similar predictive abilities for both protocols. Both protocols’ risk scores made a modest yet statistically significant contribution to explaining all-cause mortality and cardiovascular events. Moreover, when a clinical model including readily available variables (age, gender, smoking, diabetes, CV comorbidities, cholesterol, arterial pressure, anti-hypertensive treatment, hemoglobulin, phosphate, and albumin) was added to the two scores separately, then similar predictive findings were also recorded. Additionally, the time required to perform each protocol was also recorded. This study did not demonstrate any superiority of one protocol over the other regarding the detection of pulmonary congestion or the prediction of death or cardiovascular events over a 3-year period. At the same time, the 8-point protocol was performed in a shorter period of time [1.3 min (IQR 1.2–2.0)] compared to the 28-point protocol [3.0 min (IQR 2.2–5.0)]. However, it is worth noting that the 8-point protocol described by Volpicelli was not applied; instead, eight other anatomical positions were selected, which are included in the 28-zone protocol. Figure 5 illustrates the difference between the two different 8-zone scanning protocols.

### 3.2. Articles Including the Comparison of the 28-Zone Protocol with the Most Commonly Alternative Protocols Requiring Fewer Scanning Zones (8 Zone, 6 Zone, and 4 Zone) in Adult HD Patients

The following three articles involve the comparison of the full scanning protocol with the most abbreviated scanning protocols reported in the literature.

Firstly, a study published by Reisinger et al. compared the 28-zone protocol with the 8-zone, 6-zone, and 4-zone protocols [30]. This is the first study to conduct such a comparison in the literature. Particularly, 98 HD patients were included from a single dialysis unit, and measurements were performed in the emergency department. Various examiners conducted the measurements, including undergraduate students, who were prepared via an online seminar and were subsequently supervised during their first 10 measurements. The participants were mainly African Americans (84%) who presented to the emergency department with a variety of symptoms. Specifically, the most common causes for presentation were reported dyspnea (49%) and CV incidents (44%). The CV events were primarily due to either heart failure decompensation or fluid accumulation. Additionally, some patients (17%) presented with respiratory infections.

Regarding the 28-zone protocol, the presence of 15 B-lines was set as the threshold. Mild to moderate pulmonary congestion was defined as the presence of B-lines ranging from 0 to 15, whereas a count of more than 16 B-lines was classified as moderate to severe pulmonary congestion. This classification constitutes a simplification of the commonly used classification of pulmonary congestion in relation to the 28-point protocol (0–5: absence of pulmonary congestion, 5–15: mild pulmonary congestion, 16–60: moderate pulmonary congestion, and >60: severe pulmonary congestion). Concurrently, a threshold was established for the protocols requiring fewer anatomical sites scanned on the chest wall. This threshold was the number of B-lines recorded during the measurements divided by the number of zones where the measurements were taken. A threshold of 0.54 B-lines per zone was established as the threshold for moderate fluid overload, corresponding to the 15 B-line threshold in the 28-point protocol.

The participants presented a mean of 43 ± 40 B-lines during evaluation with the 28-point protocol. Statistical analysis revealed a significant correlation between the findings of the other protocols and their accuracy in predicting mild pulmonary congestion and moderate to severe congestion, respectively. Each of the abbreviated protocols, which required the assessment of fewer anatomical sites, was able to distinguish between no-to-mild and moderate-to-severe fluid overload. The patients were followed for a median of 778 ± 175 days. Forty-six patients died, of which thirty-four had moderate-to-severe fluid overload (49%), and twelve had no-to-mild fluid overload. The diagnostic accuracy among the four protocols studied was similar for detecting pulmonary congestion in HD patients evaluated in the emergency department, with no significant difference in mortality between patients with no-to-mild fluid overload and those with moderate-to-severe fluid overload over the 30-day follow-up period. However, this study has several limitations. Initially, the measurements were conducted in the emergency department, where examiners may perform the measurements quickly based on the severity of the clinical presentation. Additionally, the study was conducted at a single center. Moreover, the assessment system applied for the three abbreviated protocols was an arbitrary decision by the authors. Their main goal was to propose an alternative version that could also classify the degree of hypervolemia faster than the 28-zone protocol. However, this methodology was based on the assumption that the distribution of B-lines is symmetrical. This study presented no data referring to the mean time required for performing each protocol.

Secondly, Sonko et al. conducted a study exploring whether the 28-zone protocol can be simplified by scanning fewer zones with similar results in detecting fluid accumulation [31]. Statistical analysis was performed from 2800 LUS clips. In total, 100 HD patients underwent LUS. A strong correlation between the B-line score gained from the 28-zone protocol and the other protocols (8 zone, 6 zone, 4 zone) has been well demonstrated. Correlation coefficients of 0.92, 0.92 and 0.95 and Cohen’s Kappa values of 0.71, 0.76 and 0.74 corresponded to the 8-zone, 6-zone and 4-zone protocols, respectively. These Cohen’s Kappa values showed a satisfactory correlation with the detection of fluid accumulation severity. In addition, a new 4-zone protocol was mentioned and considered to be even more accurate, with a Cohen’s Kappa of 0.82. So, this study supports the fact that protocols with fewer scanning zones can provide important clinical information regarding to fluid status of HD patients. A new scanning 4-zone protocol was described and showed better accuracy than the other, requiring fewer scanning zones, although no further details were published.

On the other hand, Keskinis et al. recently published a study including 68 HD patients [1,11]. This study’s goal was to assess the diagnostic accuracy of hypervolemia among all the existing scanning protocols. The study took place in a single dialysis unit, and strict exclusion criteria were applied. All the participants underwent LUS measurements before and after the scheduled dialysis session. All the measurements were performed by a single operator, a nephrology trainee. This is the first study that involves pre- and post-dialysis ultrasonographic evaluation by comparing all protocols. The 28-zone protocol was considered the most informative and the most accurate. The post-dialysis Kappa values were 0.433, 0.516 and 0.433 for the comparison between the 28-zone protocol and the 8-zone, 6-zone, and 4-zone scanning protocols, respectively. Meanwhile, the pre-dialysis Kappa values were even lower. This study’s limitations are its short monitoring, the lack of patient follow up and the existence of only one examiner. Moreover, the findings are derived from single-center measurements. The authors consider the 28-zone protocol the most reliable tool for estimating DW in HD patients, although it is not feasible for daily practice. Therefore, alternative protocols may be useful for monitoring, while the 28-zone protocol can be used when more data are required.

### 3.3. Articles Including the Comparison of the Other Scanning Protocols (16-Zone, 12-Zone, and 8-Zone Scanning Protocols)

**Figure 6:**
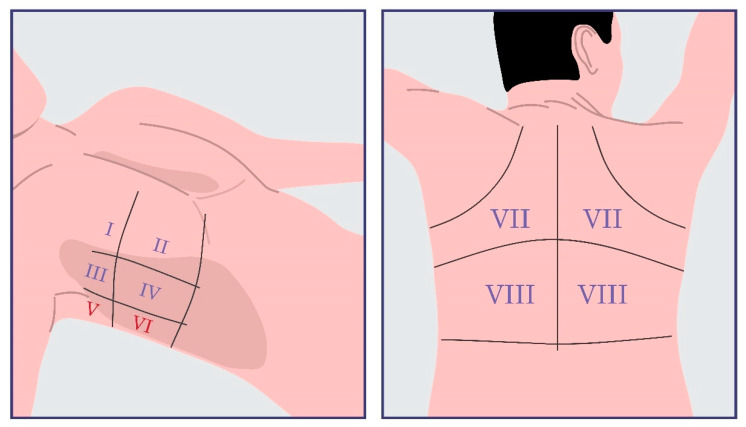
The 16-zone protocol involves eight scanning zones on each hemithorax as visualized. The following anatomical sites are included: superior medial, superior lateral, superior axillary, inferior medial, inferior lateral, diaphragmatic, upper back, and lower back. A simplified version has been adjusted by excluding the typed-in-red V and VI anatomical sites bilaterally, as depicted above.

Recently, Raz et al. published a single-center study aimed at uncovering the role of LUS in the assessment of CKD patients’ volume status [32]. Patients at various stages of CKD were included, specifically those on HD, peritoneal dialysis (PD), and non-dialysis (CKD 1–5 ND). In total, 175 participants were enrolled: 119 HD patients, 19 PD patients and 37 CKD 1–5 ND patients. All HD patients were assessed twice, before and after the dialysis session, while the rest of the participants were evaluated only once. We focused mainly on the subpopulation of HD patients due to the requirements of this review. The primary aim of the study was the correlation of the 12-zone protocol findings with physical examination results in assessing fluid overload in CKD patients. Additionally, different LUS protocols were compared (16 zone, 12 zone and 8 zone), and a correlation between LUS score change and the applied ultrafiltration was evaluated. Particularly, the 16 zone and 12 zone protocols are depicted in Figure 6. Two physicians performed the physical assessment of all participants. The measurements were performed by one of the two examiners, both of whom were trained according to the protocol of the study. Initially, the first measurements were conducted simultaneously by both examiners. In cases of disagreement, the principal investigator was responsible for determining the final result. Patients were evaluated while sitting. Initially, a 16-zone protocol was applied using both longitudinal and transverse views through the intercostal spaces. These zones included eight points on each hemithorax: upper medial, upper lateral, upper axillary, lower medial, lower lateral, diaphragmatic, upper back and lower back. However, this protocol was considered to be time consuming and difficult, particularly for patients with tunneled dialysis catheters in the jugular veins. Consequently, the protocol was adjusted to a more simplified 12-zone approach, which excluded the superior and inferior lateral zones on both sides.

Systolic blood pressure did not show a significant correlation with LUS scores in HD patients, but several other clinical data (presence of lung crackles, pleural effusion, and peripheral edema) were significantly correlated with LUS scores. Oxygen saturation in room air also showed a significantly negative correlation with LUS scores.

The accuracy of the three scanning protocols applied was compared to evaluate whether it was similar. Each zone was given a score based on the number of B-lines observed: 0 points for no B-lines or less than two, 1 point for more than three B-lines, 2 points for multiple coalescing B-lines, and 3 points for pulmonary consolidation [32]. The total score for the 12-zone evaluation ranged from 0 (indicating normal findings) to 36 (indicating severe congestion). According to these scores, participants were divided into two categories: the “low group”, representing either no congestion or mild congestion (LUS score 0–12), and the “high group”, representing moderate to severe congestion (LUS score above 13). In the whole study’s population, the low group was younger (68.5 ± 13.5 years) compared to the high group (75.6 ± 6.8 years, *p* < 0.01) [32]. The groups did not differ significantly in terms of systolic blood pressure, although in HD patients, only pre-dialysis measurements were taken into consideration to avoid post-dialytic hypotension [32]. Moreover, oxygen saturation was significantly lower in the high congestion group (96.6% ± 2.9%) compared to the low group (97.8% ± 1.5%). There was also a significant correlation between higher LUS scores and several other clinical data (presence of lung crackles, pleural effusion, and peripheral edema). On the other hand, in patients with PD, no correlation was observed between physical examination findings and LUS findings, possibly due to the small sample size of PD participants.

No significant correlation was found between ultrafiltration volume (calculated from pre- and post-dialysis weight change) and changes in LUS scores, assuming that the fluid redistribution in the human body may be a lengthy procedure. A strong correlation between the 16-zone and 12-zone scanning protocols was displayed (Pearson correlation coefficient = 0.907). This was evident across different patient groups, including HD patients, suggesting that the simplified 12-zone protocol could be a reliable alternative for the detection of lung congestion. The median 12-zone LUS score for the HD group was 7 (IQR 4–11). Additionally, a strong linear correlation was also observed between the 12-zone protocol and the 8-zone protocol when the posterior thorax fields were excluded.

This is a single-center study, limiting the broad applicability of its findings to other populations. In addition, since LUS is operator dependent, the use of only two trained physicians or ultrasound technicians helped reduce bias, but there may still be some variation in technique. The study only captured LUS and physical examination data at a single point in time, which restricts the ability to evaluate reproducibility or changes in volume status over time. Overall, the study demonstrated that LUS is an effective tool for assessing volume status in CKD patients, with strong correlations found between the LUS results and physical examination findings. The simplified 12-zone protocol proved to be just as effective as the more complex 16-zone protocol, offering a more practical option for clinical use. While blood pressure did not show a correlation with fluid overload, other physical examination indicators, such as lung crackles and edema, did.

### 3.4. Articles Including the Comparison of the 28-Zone Protocol with the Corresponding 8-Zone Protocol in Pediatric HD Patients

There is only one study in the literature that compared the 28-zone protocol with the corresponding 8-zone protocol in a pediatric population of HD patients [33]. In total, 15 patients were included. Patients’ consent was given for the assessment of anonymized data. Each participant underwent a lung ultrasound using the 28-zone protocol before hemodialysis. The 8-zone protocol, which was also used in the study by Torino et al. and not by Volpicelli et al., was also applied [22,25]. The results of this protocol were multiplied by 28/8 so that they could be compared with those of the 28-zone protocol. A strong correlation between the 28-zone and 8-zone protocols was detected (Spearman’s rho = 0.91, *p* < 0.001). In only one patient, hypervolemia (six B-lines in the 28-zone assessment) was missed during the 8-zone assessment. However, no significant differences were found in the rest of the assessments.

All the aforementioned studies are summarized in Table 1.

The validated Quality Assessment of Diagnostic Accuracy Studies (QUADAS-2) scoring system was used to assess each study according to the following variables [34]:

1. Patient Selection;
2. Index Test;
3. Reference Standard;
4. Flow and Timing.

Each variable has been rated as follows:

- Low risk of bias. The study follows established guidelines and best practices in this area.
- High risk of bias. There are substantial concerns regarding the methodology in this domain.
- Unclear risk of bias. The available information is insufficient to form a clear judgment about the quality of the study in this domain [34].

Torino’s study received a low RoB in terms of the patient selection (a random sample of HD patients), high applicability of the cohort, and low concerns for the applicability of the index test, as it used a validated LUS protocol. However, the examiners’ experience (with only two of five examiners familiar with LUS) presented a moderate concern for bias. Reisinger’s study showed a high RoB in terms of examiners’ qualifications (undergraduate students and clinical fellows), leading to concerns about accuracy. However, it showed moderate applicability as it focused on a specific ethnic group (84% African American) and took place in an emergency department setting, which could limit the generalizability to non-emergency clinical settings. Additionally, no clear information on the patient selection process (single center) presented a moderate risk of bias in patient selection. Sonko’s study raised significant concerns regarding its applicability due to being conducted at a single center, with an unclear number of examiners and insufficient information on their qualifications. Nevertheless, the results remained consistent across the different protocols. Additionally, the patient selection process was not thoroughly described, leading to moderate concerns about the generalizability of the findings. Keskinis’ findings presented a low RoB in patient selection, with strict exclusion criteria applied, but examiner bias was a concern, as only one examiner performed all measurements. The study location in a single-center dialysis unit limited the applicability of the results to broader populations. Raz’s study had a low risk of bias in terms of patient selection (inclusion of various stages of CKD) and index test applicability (use of trained examiners). However, the single-center nature of the study and the small sample size of PD patients raised concerns about the generalizability and applicability of the findings, contributing to a moderate concern for applicability. While Allinovi’s study had a small sample size, it demonstrated a low RoB in both the accuracy of the index test and patient selection. The applicability concerns were also minimal, as the study focused on pediatric HD patients in a single-center setting. However, the limited sample size and single-center nature of the study raised questions about how broadly the results could be applied. A summarized risk of bias for each individual study is depicted in Table 2.

## 4. Discussion

In total, 688 adult HD patients were included in the first five studies. The heterogeneity observed in these studies can be interpreted in several ways as follows: the presence of different inclusion–exclusion criteria, varying ethnicities, different comorbidities, and the different methodologies applied to each study. Specifically, each study shows significant differences compared to the others regarding when the measurements were taken, who performed the measurements, where they were conducted, and the patient’s general status at the time of examination. There is a variety of populations studied (Caucasians or African Americans) because every study was conducted at a different single center. The number of examiners also differs since some studies were conducted by a single examiner, while others involved two, five, or even more examiners. The existence of a sole examiner entails subjectivity in the assessment of the results. This bias grows further when the same study includes both pre- and post-dialysis measurements, as in the study by Keskinis et al. since the same examiner is aware of the initial ultrasound findings and could potentially influence the post-dialysis evaluations [1,11]

Initially, Torino et al. tried to introduce a simplified version of the 28-zone protocol by comparing it to an 8-zone protocol [25]. The duration of the 28-zone protocol ranged from 2.22 to 5.00 min, with a median time of 3.05 min [25]. These findings were a little longer than those published by Jambrik et al. (2.8 ± 0.4 min), who were the first to describe this protocol [24]. At first glance, the duration of the examination may not seem particularly long, but it should be taken into consideration that every nephrologist manages many HD patients during daily clinical practice, so integrating the 28-zone protocol is quite difficult. Meanwhile, the 8-zone protocol implementation lasted about 1.35 min (IQR 1.16–2.00), which is almost half the time required for the 28-zone protocol. Furthermore, the existence of five examiners, three of whom were not quite familiar with the technique, highlights the fast learning curve of the technique [25]. However, not using the widely accepted 8-zone protocol, as proposed by Volpicelli, was an important variation in this study [27]. Instead, eight different anatomical sites, as illustrated in Figure 4, were used to draw immediate conclusions. In general, the 8-zone protocol does not seem to be superior to the 28-zone protocol, although it provides similar clinical information in many cases [25]. Both protocols can be useful in daily clinical practice, depending on the severity of hypervolemia and lung congestion. For instance, if a patient presents with mild or no hypervolemia, applying the 8-zone protocol may be adequate, but the 8-zone protocol may provide uncertain findings in patients with moderate lung congestion. In these cases, the 28-zone protocol may be more useful for evaluating the severity of lung congestion.

Additionally, three studies have compared the 28-zone protocol with the most commonly reported protocols in the literature, which are the 8-zone, 6-zone, and 4-zone protocols. Each of these studies took place at a single center, which was different from study to study. Moreover, patients of the same ethnicity were not included in every study, and the studies were not performed under the same conditions. For example, Reisinger et al. focused on HD patients who arrived at the emergency department with different symptoms, such as dyspnea [30]. This study was based on the work published by Buessler et al., which compared the 28-zone protocol with the most well-known abbreviated versions (8-zone, 6-zone, and 4-zone protocols) in patients suffering from heart failure [35]. Reisinger’s study was to evaluate whether they could replace the 28-zone protocol in daily clinical practice. Furthermore, this study noted that certain anatomical sites on the chest showed a greater correlation with increased B-line presence. The presence of 15 B-lines in the 28-zone protocol is known to show moderate hypervolemia. A threshold was proposed for the other three protocols, corresponding to the 15 B-lines of the 28-zone protocol [30]. Specifically, this threshold was defined as 0.54 B-lines per zone in the other three protocols [30]. In fact, this threshold was established by dividing the number of B-lines corresponding to moderate hypervolemia (15) by the total number of scanning zones (28). This threshold has been proposed for use in other protocols with fewer scanning positions. For example, the hypervolemia threshold for the 8-zone protocol is 4–5 B-lines, which is derived by multiplying 8 (the total number of scanning positions) by 0.54. This methodology provides a common basis for comparison across all scanning protocols in terms of hypervolemia assessment. In practice, 0.54 B-lines per zone roughly corresponds to 1 B-line per 2 zones, or more generally, the hypervolemia threshold can be estimated by dividing the total number of scanning positions for each protocol by 2.

However, this correlation is not accurate because it relies on the assumption that B-lines are symmetrically distributed, which seems not to be correct [30]. Variations in the criteria used to define hypervolemia may lead to inconsistent results, potentially complicating the interpretation of findings and influencing treatment decisions. The need for standardized protocols is underscored, and a unified classification system is suggested to ensure more reliable and reproducible assessments across clinical settings.

Conducting the study in an emergency department setting presents certain limitations, such as the need for immediate measurements. This is the only study conducted under these circumstances, which is another factor complicating the classification and the comparison among the studies. The presence of various examiners, including undergraduate students, creates a particularly heterogeneous group of examiners. This study included most examiners, and the exact number is not known. The participants’ mortality was followed up for 30 days, and no difference among the four protocols was detected. The diagnostic accuracy of the four protocols was also similar. The findings of this particular study must always be assessed, considering that it took place in an emergency department, where some patients even presented with dyspnea. In fact, the participants had a mean 43 ± 40 B-line score when assessed using the 28-zone protocol, a finding corresponding to severe hypervolemia. Therefore, this study presented similar findings in HD patients with acute symptoms of severe hypervolemia rather than in stable HD patients without obvious signs of hypervolemia, despite the presence of fluid in the extracellular lung tissue. Conclusively, this study advocates that all four protocols have similar accuracy in HD patients with severe pulmonary congestion, indicating that scanning all 28 zones may not be necessary.

The second single-center study, which assessed the correlation among the 28-zone, 8-zone, 6-zone, and 4-zone protocols, included 100 HD patients [31]. All participants were admitted to the acute care center and underwent the 28-zone protocol. Subsequently, all three abbreviated protocols showed similar findings to the 28-zone protocol when tested using linear correlation and discriminant analysis. However, a new 4-zone protocol had even better accuracy compared to the other abbreviated protocols. Unfortunately, this study does not mention the exact anatomical sites used in the new 4-zone protocol.

On the contrary, Keskinis et al. published a study that included HD patients who came for their scheduled mid-week session [1,11]. The aim was the detection of the participants’ average fluid status. Measurements were performed by a single examiner, who was trained by doctors in an intensive care unit, before and after dialysis. Very strict exclusion criteria were applied in the study to ensure that the presence of B-lines could not attributed to heart failure or any other comorbidities. All measurements were conducted 30 min before and 30 min after the end of dialysis. All protocols were assessed pre- and post-dialysis. The 8-zone protocol was evaluated using the Volpicelli protocol and not the alternative mentioned above by Torino et al. [25,27]. The total duration of all four measurements combined with the written recording of B-lines for each protocol lasted approximately 10 to 12 min per patient. This study is the first to show that the three abbreviated protocols did not demonstrate significantly higher accuracy compared to the 28-zone protocol. This may be explained due to the strict exclusion criteria applied. Ten patients were diagnosed with hypervolemia according to clinical criteria, a small proportion of whom were identified as hypervolemic using the 8-zone, 6-zone, and 4-zone protocols. The authors do not believe that any shorter version of the protocol can replace the 28-zone protocol in assessing DW in HD patients. Keskinis et al. [1] showed that shorter protocols have similar accuracy in detecting lung congestion when it is clearly apparent. For example, only two patients were considered hypervolemic according to the 8-zone and 4-zone protocols, presenting post-dialytic 62 and 72 B-line scores, respectively. The 6-zone protocol indicated three patients as hypervolemic, including the two patients mentioned above. All three patients still presented ultrasonographic findings of indicative lung congestion and hypervolemia after the dialysis session.

The study by Raz et al. also constitutes a single-center study that first introduced the use of lung ultrasound by examining 16 and 12 sites on the thoracic wall [32]. The study compared these two protocols with the 8-zone protocol. Unfortunately, this study did not include the 28-zone protocol, which was the first ever described for HD patients and has been the most extensively evaluated in many studies. The absence of this protocol limits the findings of the study in terms of comparison with other studies. However, a significant correlation was found between the physical examination (oxygen saturation, crackles upon lung auscultation, lower extremity edema, pleural effusion) and the ultrasonographic findings in HD patients, regardless of which protocol applied [32]. This finding contrasts with the results of the LUST (Lung Water by Ultrasound-Guided Treatment to Prevent Death and Cardiovascular Complications in High-Risk ESRD Patients with Cardiomyopathy) trial, which emphasized the presence of lung congestion in HD patients without apparent clinical signs of fluid accumulation [16]. Therefore, clinical examination should not be skipped or underestimated [32]. On the other hand, arterial hypertension did not show a significant correlation with ultrasound findings, indicating the complex pathophysiology associated with hypertension in HD patients [32,36]. Furthermore, the study did not find a correlation between the applied ultrafiltration volume and the reduction in B-lines [32]. This observation is probably attributed to the time needed for fluid redistribution in the body [32]. In contrast, the study by Keskinis et al. demonstrated that the application of 2.438 ± 0.229 L of ultrafiltration led to a reduction in B-lines by 5.96 ± 2.19 after 30 min following the completion of the dialysis session [11]. On the other hand, Raz et al. did not involve the exact timing of the measurements, whether they were taken before or after the dialysis session [32].

Several studies have evaluated the 28-zone, 8-zone, and 4-zone ultrasound protocols in various settings, including septic patients. One notable study was a prospective, single-blind, cross-sectional design involving 44 septic individuals. In this study, transpulmonary thermodilution, the gold-standard method for measuring extravascular lung water, was used for comparison [37]. The findings revealed that the 28-zone protocol was more specific and sensitive in detecting lung congestion in critically ill septic patients than both the 4-zone and 8-zone protocols. The 28-zone protocol was particularly more accurate in diagnosing lung congestion in patients with acute respiratory distress syndrome (ARDS) when compared to the shorter protocols [37]. A separate study by Mayr et al., involving 50 critically ill patients, assessed the performance of the 28-zone and 4-zone protocols [38]. While both protocols showed similar effectiveness in identifying severe pulmonary edema, the 28-zone protocol demonstrated a slight advantage in detecting mild to moderate lung congestion. However, it was also noted that the 28-zone protocol required a longer time for evaluation compared to the 4-zone protocol [38].

The results in children are quite optimistic, like in some of the aforementioned adult studies, suggesting that the 8-zone protocol is closely correlated with the 28-zone protocol, providing almost the same predictive power [33]. Loutradis et al. were the first to propose an alternative scanning protocol in children because an overlap, attributed to transducer size, might happen between the different scanning sites when the 28-zone protocol is performed [39]. The only existing study, which evaluated an alternative approach in children, supports the use of the 8-zone LUS for DW estimation in children on dialysis, targeting to decrease the number of scanning zones required [33]. This idea should be further validated in order to assess fluid accumulation in children, taking into consideration more factors like age and body surface area.

LUS is increasingly recognized as a valuable diagnostic tool for evaluating respiratory conditions in neonates and children [40]. B-lines are associated with various lung diseases, including edema, interstitial lung disease, infections, and congestive heart disease. The distribution and pattern of B-lines can help in diagnosing these conditions and assessing their severity. In neonates and children, scattered or isolated B-lines may be normal, but an increased number often indicates more severe respiratory issues [40].

LUS can distinguish between different respiratory conditions, such as transient tachypnea of the newborn (TTN) and respiratory distress syndrome (RDS), based on the B-line patterns observed. Several scoring systems have been developed to quantify B-lines, and some are already showing promise in clinical applications. These include the following:

- Surfactant administration in preterm neonates, based on B-line patterns;
- Evaluation of fluid overload in pediatric dialysis patients;
- Prediction of complications, like bronchopulmonary dysplasia (BPD).

While technical factors, such as the type of probe used and the experience of the operator, can influence B-line detection, LUS offers a potential point-of-care solution for diagnosing lung conditions in children. However, further studies are needed to standardize B-line quantification and confirm its role in clinical practice.

Several LUS scanning protocols for quantifying B-lines are outlined as follows:

- Two-zone scan. This is used in critically ill patients with cardiogenic pulmonary edema, focusing on the anterior hemithorax to identify diffuse B-lines [41].
- Four-zone scan. This is applied in pediatric cardiac surgery to assess pulmonary congestion and differentiate between “white lung” (confluent B-lines) and less severe B-line patterns [42].
- Six-zone scan. This is common in neonatal intensive care units (NICUs) to assess loss of lung aeration, with a scoring system from 0 (normal) to 3 (complete loss of aeration) [43].
- Eight-zone scan. This is used in emergency departments to diagnose interstitial syndrome. A positive exam requires ≥3 B-lines in two or more areas [44].
- A simplified 8-site score. This is used in nephrology and dialysis settings to evaluate fluid overload, with hypervolemia defined by ≥3 B-lines in specific sites [33].
- Twelve-zone scan. This is applied in nephrology and dialysis, where euvolemia is defined by <5 B-lines and hypervolemia by >10.5 B-lines [45].
- Fourteen-zone scan. This is used in cardiology and nephrology to assess fluid overload, with moderate hypervolemia defined by ≥10 B-lines [46,47,48,49].
- Twenty-eight-zone scan. This is a comprehensive method used in cardiology and nephrology, where euvolemia is defined by ≤5 B-lines and moderate hypervolemia by ≥15 B-lines [24,48,49].

However, these pediatric-specific protocols have not yet been directly compared with one another or with those discussed in the current study. The 14-zone protocol involves scanning bilateral areas from the second to the fifth intercostal space on the right side and from the second to the fourth on the left side. B-lines are quantified in 14 intercostal positions for patients under 20 kg and in 28 positions for those over 20 kg [46,48]. Incorporating these additional scanning protocols and findings into future research could enhance the understanding of fluid management in pediatric patients, especially those undergoing dialysis. This will ultimately contribute to better clinical practices and improved patient care.

Taking into account all the data presented, it is concluded that the 28-zone protocol provides more information regarding the assessment of lung volume in HD patients compared to abbreviated protocols. However, this does not mean that shorter protocols have no practical application. Based on our experience, we believe that shorter protocols can be applied in the following cases:

- Initial Screening with Fewer Scanning Zones. The examiner may begin by evaluating fewer scanning points on the chest wall, and if ultrasound findings indicate hypervolemia, then proceed with the 28-point protocol.
- Recent Full Assessment. If the patient has recently been assessed using the 28-point protocol and no lung congestion was detected, they may be monitored using a more concise protocol, without unnecessarily repeating the full 28-point protocol.
- Clear Evidence of Lung Congestion. If the patient already presents clear signs of lung congestion from the abbreviated protocols, there is no clinical question to address, apart from possibly a more detailed classification/assessment of the fluid overload.

The application of abbreviated protocols in these contexts allows for efficient patient monitoring while avoiding unnecessary repetition of detailed assessments.

Diversity is observed among the examiners who performed the LUS measurements. There is no study that was conducted by the same number of examiners with similar experience. This finding highlights the need for the establishment of a certified training program in LUS for nephrologists so that each examiner’s experience can be officially certified. Furthermore, the heterogeneity in the experience/knowledge of the examiners represents a significant limitation in our study. Another considerable finding is the significant heterogeneity observed regarding the classification of hypervolemia in the protocols that required fewer scanning zones. No reference was included in the studies published by Torino et al., Sonko et al., and Allinovi et al. related to the classification of hypervolemia in the fewer scanning zone protocols [25,31,33]. However, in the other studies, this classification is recorded, which is entirely different from one study to another [1,11,30,32]. The monitoring of asymmetrical B-line presence has been well established in the literature, but the pathogenesis remains unknown [30,50]. Donadio C et al. have shown that the distribution of B-lines is not homogeneous among different scanning zones since 2015. Specifically, there are anatomical sites where the presence of B-lines is more common in HD patients presenting with pulmonary congestion than in other sites [50].

QUADAS-2 criteria have also confirmed the aforementioned limitations, notably the variability in study quality, inconsistencies in the reporting of methodological details, and the small sample sizes observed in some studies. These factors complicate the ability to draw definitive conclusions regarding the diagnostic accuracy of LUS in HD patients. To address these challenges, future research should prioritize more rigorous study designs, incorporating clear and standardized protocols for patient selection, examiner qualifications, and reference standard validation. Furthermore, multicenter studies that encompass a wide range of patient populations and ensure consistency in diagnostic protocols are essential to improving the generalizability and applicability of the findings.

## 5. Conclusions

Since nephrologists’ aim remains the integration into daily clinical practice of a feasible protocol requiring fewer scanning zones, it is essential to involve in the LUS protocol the anatomical sites where hypervolemia is most commonly recorded. Hypervolemia is not detected symmetrically in HD patients via LUS. Moreover, it is not entirely clear which positions on the chest wall show a more prominent presence. We acknowledge that the inclusion of only six studies in our review may limit the generalizability of our findings. However, no additional studies meeting the defined inclusion criteria were identified during the review process. Therefore, we believe that all relevant research available at the time has been considered. Furthermore, the studies included in our analysis exhibited substantial methodological variability and differences in primary outcomes. Consequently, we advise that our findings be interpreted with caution, given these limitations. The conduct of a multicenter study including HD patients from different dialysis units could probably give us a clearer answer. Validation in multicenter trials is a crucial next step for confirming the effectiveness and generalizability of the abbreviated protocols across diverse patient populations and clinical settings. These trials would help to further assess the accuracy, efficiency, and feasibility of these protocols in real-world practice. Until such studies are conducted, it is important to remember that the 28-zone protocol undeniably offers detailed clinical data and classifies our findings, despite its time-consuming nature. In cases of moderate hypervolemia, the nephrologist should perform a more thorough examination of the chest wall, while in situations where there are no clinical doubts, shorter protocols may be adequate to provide the necessary information.

## Figures and Tables

**Table 1:** The main clinical parameters of the included studies are recorded to make their findings clearer.

No.	Reference	Number of HD Pts	Mean Age (Years)	Scanning Protocols	Ethnicity	Number of Examiners	Location of the Measurements Conducted	Measurements’ Duration	Limitations	Conclusion
1	Torino et al. [25]	303	66 ± 13	28 zone 8 zone	Italian	5	Hemodialysis unit	28 zone: 3.0 min (2.2–5.0); 8 zone: 1.3 min (1.2–2.0)	Modified 8-zone protocol, with some sites adapted from existing protocols	The 28-zone and 8-zone protocols show equivalent predictive value for outcomes.
2	Reisinger et al. [30]	98	59 ± 15	28 zone 8 zone 6 zone 4 zone	Black or African American race (84%) Hispanic or Latino (5%)	Multiple examiners (unknown number) Fellows and postbaccalaureate students	Emergency department	Not mentioned	All measurements were conducted in the emergency department Single-center study	All protocols had comparable hypervolemia detection rates and mortality outcomes.
3	Sonko et al. [31]	100	Not mentioned	28 zone 8 zone 6 zone 4 zone (×2)	Not mentioned	Not mentioned	Acute care treatment	Not mentioned	Single-center study	All scanning protocols showed similar correlations, with the new 4-zone protocol offering better accuracy than existing abbreviated versions.
4	Keskinis et al. [1,11]	68	65.01	28 zone 8 zone 6 zone 4 zone	Greek	1	Hemodialysis unit	The total duration for all four protocols was approximately 10 to 12 min per patient	One examiner performed all measurements Single-center study	The 28-zone protocol was more effective in detecting hypervolemia.
5	Raz et al. [32]	119	69.5 ± 12.8	16 zone 12 zone 8 zone	Not mentioned	2	Hemodialysis unit	Not mentioned	The physical examination is operator dependent Single-center study	Physical examination is valuable in detecting hypervolemia, even though hypertension showed no correlation with it.
6	Allinovi et al. [33]	15	13.6 (4.1–16.4)	28 zone 8 zone	Not mentioned	Not mentioned	Hemodialysis unit	Not mentioned	Single-center study	The 8-zone score accurately detected hypervolemia in the pediatric population.

**Table 2:** All the studies included in our review have been evaluated using the validated Quality Assessment of Diagnostic Accuracy Studies (QUADAS-2) scoring system.

	Risk of Bias (Low/High/Unclear)			
Study	Patient Selection	Index Test	Reference Standard	Flow and Timing
Torino et al. [25]	Low	Low	Unclear	Low
Reisinger et al. [30]	Unclear	Unclear	Unclear	Unclear
Sonko et al. [31]	Unclear	Unclear	Unclear	Unclear
Keskinis et al. [1,11]	Low	Low	Unclear	Unclear
Raz et al. [32]	Unclear	Low	Unclear	Unclear
Allinovi et al. [33]	Low	Low	Unclear	Unclear

## Data Availability

All data are available upon request from the corresponding author.
